# Experiences of Telenursing in Overcoming Challenges and Applaying Strategies by COVID-19 Patients in Home Isolation: Qualitative Study in Primary Care

**DOI:** 10.3390/healthcare11142093

**Published:** 2023-07-22

**Authors:** Glòria Tort-Nasarre, Anna Espart, Paola Galbany-Estragués, Bruna Álvarez, Martí Subias-Miquel, Maria Romeu-Labayen

**Affiliations:** 1 SAP ANOIA, Gerència Territorial Catalunya Central, Institut Català de la Salut (ICS), 08700 Igualada, Spain; 2 Department of Nursing and Physiotherapy, Faculty of Nursing and Physiotherapy, University of Lleida, 25198 Lleida, Spain; anna.espart@udl.cat; 3 AFIN, Research Group and Outreach Centre, Autonomous University of Barcelona, 08193 Cerdanyola del Vallés, Spain; pgalbanye@ub.edu (P.G.-E.); mariabruna.alvrez@uab.cat (B.Á.); marti.subias@pssjd.org (M.S.-M.); mariaromeu@ub.edu (M.R.-L.); 4 Development of Healthy and Sustainable Organizations and Territories (DOTSS), 25001 Lleida, Spain; 5 Research Group of Health Care (GRECS), Institute for Biomedical Research, Dr. Pifarré Foundation, IRBLleida, 25198 Lleida, Spain; 6 Department of Fundamental and Medical-Surgical Nursing, School of Nursing, University of Barcelona, 08907 L’Hospitalet de Llobregat, Spain; 7 Parc Sanitari Sant Joan de Déu, Camí Vell de la Colònia, 25, 08830 Sant Boi de Llobregat, Spain; 8 Department of Public Health, Mental Health and Mother-Infant Nursing Faculty of Medicine and Health Sciences, University of Barcelona, 08907 L’Hospitalet de Llobregat, Spain

**Keywords:** COVID, lockdown, telenursing, nursing, primary health care, qualitative research

## Abstract

During the first wave of the COVID-19 pandemic, there was a significant increase in the use of telenursing to provide care for patients at home. However, the quality of the patient experience when nurses rely on technology instead of personal contact has not been thoroughly investigated. This study aimed to understand the perspectives of COVID-19 patients in home isolation who received telenursing from primary care nurses during the initial phase of the pandemic. A qualitative study was conducted that employed purposive sampling and involved semi-structured interviews via videoconference with fourteen COVID-19 patients from two primary health centers in Catalonia (Spain). Thematic analysis was used, and the study adhered to the COREQ checklist. The findings revealed three themes related to the challenges faced by COVID-19 patients in home isolation: physical symptoms, emotional and social difficulties, and a lack of information. Three themes emerged regarding the strategies patients employed to overcome these challenges and the role of nurses: self-care, emotional support, and personal commitment. The patients reported having achieved strategies to improve their physical, psychological, and situational well-being despite the unprecedented situation. The study highlights that telenursing is a valuable resource for delivering patient-centered care, which could lead to changes in organisational policies and the development of best clinical practices.

## 1. Introduction

Primary care has had a very important role in the response to the COVID-19 pandemic. In Spain’s Catalonia region, as in many places around the world, primary has become the main setting for diagnosing and following patients with mild or moderate symptoms and those who have been discharged from the hospital [1]. The urgent need for an alternative to in-person visits to avoid the risk of new COVID-19 infections rapidly intensified the use of telehealth in primary care [2,3]. Given the different conditions and care needs of patients with COVID-19, continuity of care for these patients was important to facilitate treatment, improve the disease and control its complications [4]. In particular, there was an unprecedented increase in telenursing to care for COVID-19 patients at home during the first wave of the pandemic. Telehealth has become a valuable option that improves the accessibility and quality of medical and nursing care. In general, patients’ experiences with telenursing have been mostly positive, as it offers convenience, accessibility and personalised care, but it is worth mentioning some inherent limitations, such as the difficulty of emotional connection and the impossibility of performing certain physical procedures [5,6,7]. Hence, telehealth was an alternative in patient care during this period in the context of primary care, but the COVID-19 patients’ experiences have not been investigated when nurses used such technology in place of personal contact, which needs further research. Understanding the experiences of the support or advice received by the nurse, along with the benefits and difficulties in a non-face-to-face system, can be very useful to advance in telenursing. Undoubtedly, more research, qualitative as well as quantitative, is needed on the integration of telenursing services in primary care and, more specifically, in supporting patients.

## 2. Background

A large proportion of COVID-19 patients have not required hospital admission, thus increasing the workload of primary care centers. Primary care nurses have played a key role in the identification, isolation and management of COVID-19 patients [8] around the world. Telenursing has made it possible to provide continuous care to COVID-19 patients who do not need to be hospitalised [9]. 

Telenursing is defined as the delivery, management and coordination of care and services via telecommunication technologies in the domain of nursing [10] to provide ongoing monitoring, support and aftercare to patients from a distance. Telenursing includes symptom management, monitoring, guidance, education and the recording of patient information. Telenurses give personal advice, promote self-care, triage health problems and refer callers to the appropriate level of health care [11]. Additionally, telenurses must have a wide range of nursing and medical knowledge and are expected to be sensitive and composed [12].

The main signs and symptoms of COVID-19 include fever, dry cough, shortness of breath, fatigue, muscle and body aches, sore throat, loss of taste and smell, nasal congestion and discharge, and headache. These symptoms can vary in intensity and present differently in each person. About 10–20% of patients with COVID-19 may require hospitalisation and medical support due to severe symptoms. The health needs of patients affected by COVID-19 have varied, as has their ability to receive and apply useful information to promote their own health. Many patients have felt a lack of knowledge and guidance about self-care, a lack of understanding of symptoms, confusion when looking for information online, fear, uncertainty and restlessness. Moreover, the stress felt by these patients has affected their physical and mental health and their ability to care for themselves [13]. 

Taking a closer look at telenursing during the first wave will help us understand how telenursing contributed to managing the disease and to patients’ self-care when they were at home in isolation. It is also important to know this point of view and perspective in order to improve or implement this model of care that warrants evaluation, and it tells a story about what has been implemented in nursing practice in response to the service delivery problems exposed by the COVID-19 pandemic.

Qualitative research about the experiences of nurses during COVID has provided valuable information [14,15], as has quantitative research about the clinical and psychological impact of patients affected by COVID worldwide [16]. However, there are few qualitative studies that describe the experiences of patients who have recovered from COVID-19 in home isolation [17,18] and, more specifically, who have received telenursing care [19]. 

The role of telenursing in the management of the chronic patient has been studied with the aim of evaluating patient safety, effectiveness and efficiency [20], but there is a lack of studies with a patient-centered approach in a non-face-to-face system such as telenursing [9].

A broad view of this experience can help researchers and policymakers to better evaluate and understand this health crisis in order to adapt health care and the primary care model—including telenursing—to meet future crises. It can also be useful to better understand aspects that guarantee an adequate implementation of this non-face-to-face care modality but, at the same time, focus on the patient. This qualitative study aimed to understand the perspectives of patients who participated in a telenursing intervention in terms of the quality of nursing care, communication, emotional support and clinical outcomes and identify the utility of the telenursing intervention for managing nursing work in the COVID-19 primary care context on young to middle-aged patients in home isolation.

## 3. Methods

### 3.1. Design

A qualitative descriptive study was conducted to determine the problem according to the individual experiences of the participants [21]. Qualitative research is focused on making sense of lived, observed phenomena in a specific context with specifically selected individuals [22]. Constructivism makes it possible to describe and interpret social and educational phenomena and to be interested in the study of the meanings and intentions of human actions from the perspective of the social agents themselves [23]. This approach must allow us to understand reality as the researched subjects see it, that is, through discovering the meaning and meaning they give to their experience [24].

### 3.2. Sample and Setting

The participants were patients with mild or moderate symptoms of COVID-19 who were recovering at home during strict confinement and who participated in a telenursing intervention at Catalonia’s primary care centres from April to July 2020. The patients were selected by purposive sampling [25] to incorporate the greatest diversity of participants possible so that we could analyse a wide range of experiences until data saturation was reached [26,27]. The primary care centers provided us with lists of patients obtained through the nurses at the primary care centers who followed up on COVID-19 patients. We contacted potential candidates in order while making some adjustments to ensure diversity in age, sex, and marital status. The inclusion criteria were as follows: being age 18 or older, having been diagnosed with COVID-19 with a positive PCR test, having recovered from COVID-19 in home isolation, having received telecare from primary care nurses while in home recovery, being originally from Spain, and being fluent in Spanish or Catalan. The exclusion criteria were as follows: having cognitive impairment and having been admitted to a hospital to recover from COVID-19. Candidates were contacted by telephone and invited to participate. As a result, fourteen participants were interviewed out of a sample of 42 patients. 

### 3.3. Intervention

Clinical follow-up for COVID-19 patients in home care was conducted via telenursing. The main objectives were to track patients’ physical symptoms, their knowledge and concerns about COVID-19, the prevention measures that they were undertaking, and the psychological impact of their illness. Nurses received initial training on how to care for COVID-19 patients via telenursing and later received updates as protocols changed. The intervention began on the day of diagnosis and continued until the patient was discharged from care. During this period, patients received a daily phone call from a nurse, in which they were invited to describe their experiences and the challenges they faced during their recovery in home isolation. On a daily basis, the nurses recorded information about the patient’s clinical course to ensure optimal follow-up. In particular, they noted down warning signs that could indicate the need for hospitalisation.

### 3.4. Data Collection

Data were collected through semi-structured interviews. Team member 1 and team member 7 jointly designed the interview guide to cover themes identified in the literature about patients with COVID and in the researchers’ own clinical experience (Table 1).

The interviews were carried out from May through July 2020 by Team members 1, 4 and 7. The interviews, recorded with the participant’s permission, took place using Teams videoconferencing software. Videoconferencing was chosen because it was a valid option during the lockdown declared by the Spanish government. This method allowed for seeing the patients’ faces and recognise them.

The interviews lasted between 30 and 45 min and took place after participants were discharged from care. We suggested that participants conduct the interview from a quiet place in which they would not be interrupted. Participants’ confidentiality was protected by giving them pseudonyms. The voice files and transcriptions were encrypted and stored on a computer protected with an encrypted password. The interviews were performed and transcribed in Catalan or Spanish. Later, the transcribed interviews were returned to the participants for their approval. All of them accepted the content of the interview.

### 3.5. Data Analysis

We conducted a thematic analysis [28] by ATLAS ti ®vs 9 support. We followed these steps to conduct the analysis: (1) Listen to recordings and enter transcripts into the software. (2) Segment the meaning units within the transcribed interviews. (3) Group meaning units into themes; the inclusion of meaning units in each theme was discussed until a consensus was reached. (4) Provide a definition for each theme and identify relationships between them. (5) Group the themes into dimensions.

### 3.6. Rigour and Quality Criteria

The trustworthiness of data was determined by credibility, dependability, conformability and transferability [29]. Credibility was achieved through analyst triangulation, constant revisions, and evaluations of themes, subthemes and units of analysis, which reinforced the validity of the data [30]. Transferability was confirmed by providing sufficient detail to be applicable to other settings and populations. Reliability was established by demonstrating the consistency and reproducibility of the findings, with a third investigator reviewing the research process. Confirmability was achieved by making reflexive efforts to avoid bias and maintaining a transparent description of the research steps. 

The research team has experience in qualitative research and resolving disagreements by consensus, and complied with the Consolidated Criteria for Reporting Qualitative Research (COREQ) [31], with constant revisions made on the thematic analysis process.

### 3.7. Ethical Considerations

The study was approved by the University Institute for Research in Primary Care (IDIAP) Jordi Gol i Gurina ethics committee (Code 20/113-P). The principles of the Declaration of Helsinki were followed. Participants received oral and written information explaining that their participation was voluntary and that they could withdraw from the project at any time. All participants provided informed consent. The interviews were anonymised by identifying each participant with an alphanumeric code in adherence with Organic Law 3/2018 on Personal Data Protection.

## 4. Findings

Fourteen COVID-19 patients who received telenursing intervention participated in the study (Table 2).

**Figure 1:**
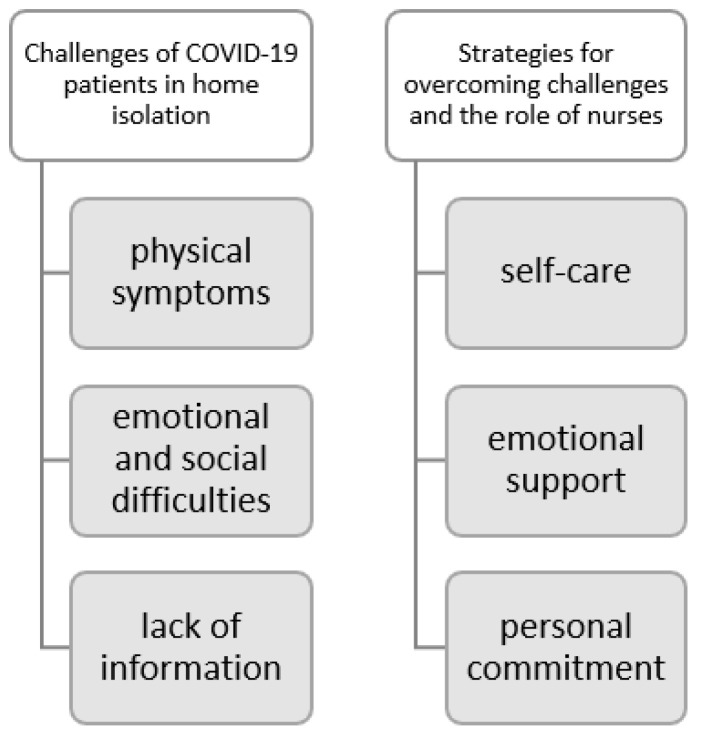
Dimensions and themes.

The results of the present study were based on two dimensions, which were subdivided into six themes that emerged from the thematic analysis (Figure 1). Three themes emerged in the “Challenges of COVID-19 patients in home isolation”: (1) physical symptoms, (2) emotional and social difficulties and (3) lack of information. Three themes emerged in the “Strategies for overcoming challenges and the role of nurses”: (4) self-care, (5) emotional support and (6) personal commitment. 

### 4.1. Challenges of COVID-19 Patients in Home Isolation

#### Physical Symptoms

First, the participants explained to the interviewers how they felt physically during the isolation period. They described a wide variety of physical symptoms, including respiratory symptoms, inflammation, fever, weakness, headache, anosmia and ageusia, ranging in severity from mild to moderate. Some participants had very mild symptoms:

*I didn’t have very strong or very clear symptoms. I had a fever for one day and later I lost my sense of smell.*

*(P14)*

Others described more generalised symptoms of moderate intensity:

*I had headache, tinnitus, itching all over my body, fatigue, loss of taste, vertigo, blurred vision, nausea, a lot of tiredness, no appetite for food, nosebleed and major gingivitis.*

*(P2)*

The variety of symptoms of the participants revealed that headache, fever, tiredness and loss of smell were the most common. We saw no link between how patients described their experiences of home isolation and the number or severity of their symptoms. That is, participants who were sicker were not more likely to describe a poor experience of home isolation. 

**Figure 2:**
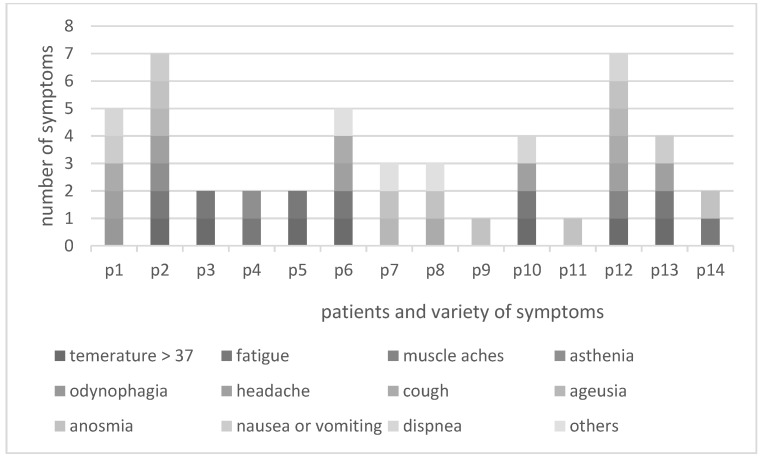
Symptoms of the participants.

Figure 2 shows the variety of symptoms of the participants, revealing that headache, fever, tiredness and loss of smell were the most common. We saw no link between how patients described their experiences of home isolation and the number or severity of their symptoms. That is, participants who were sicker were not more likely to describe a poor experience of home isolation. 

### 4.2. Emotional and Social Difficulties

The societal lockdown brought about by the first wave of the pandemic, combined with their own COVID-19 diagnosis, made many of the participants feel vulnerable and dejected.

*I felt very vulnerable. Everyone who wanted to lend me a hand, I rejected them. You don’t really know how to face your personal conflict.*

*(P13)*

They also expressed fear of infecting family members or dying.

*You have really difficult moments in which you start thinking, “If I infect someone, my family, if something happens, if things get complicated”. All kinds of thoughts come into your head.*

*(P10)*

In other cases, being connected to others allowed participants to have a more positive experience and overcome social isolation:

*While you’re sick, your people are concerned and they ask you a lot [how you’re doing] and that’s fulfilling.*

*(P10)*

Some participants also reported feeling that others blamed them for being infected by COVID-19.

*It was a pretty traumatic experience. Little support. You feel like a criminal, it’s the feeling I had. They criminalise the fact that you become infected as if you had invented the virus. It wasn’t my intention to get infected.*

*(P5)*

#### Lack of Information

Another pattern that arose in the data (despite the fact that we did not ask about it directly) was the difficulty that many participants had in obtaining accurate information about the virus and their own clinical course.

*It’s an experience of a lot of misinformation: Nobody informs you well. All the protocols change every day. Nobody tells you what to do and what not to do. Between CatSalut [Catalan health department] and the CAP [primary care centre] there is no communication.*

*(P13)*

This lack of information led participants to feel rejected and unsupported, despite the nursing telecare that they received.

*They [the nurses] do support you, and they monitor you, and you feel accompanied. But if you see that when you ask a health professional about the virus out of curiosity, and they don’t know how to answer, it makes you feel a little more alone.*

*(P4)*

Thus, the participants reported uncertainty not only about the health situation but also about the ability of their health professionals to manage their illness.

*Since there is a lot of ignorance in general about this virus, you see that there are health professionals who don’t want to take a risk when it comes to telling you things. And that creates mistrust.*

*(P11)*

### 4.3. Strategies for Overcoming Challenges and the Role of Nurses

Most participants found their situation to be complex and stressful. However, telephone calls from their nurse served to promote participants’ self-care, reduce their emotional difficulties and encourage their personal commitment to overcome the illness.

#### 4.3.1. Self-Care

Participants described learning to take care of themselves and improve their well-being. Many of them received tips on covering basic needs such as diet, physical exercise, and rest:

*I asked the CAP [primary care centre] if I could do a bit of exercise to stay in shape and they said no, that I was recovering, that my body was fighting the virus, and that I had to rest.*

*(P2)*

They explained the advice received on compliance with the prescribed medication.

*They called me to take the pills, to be calm and to take Ventolin and Paracetamol for a headache.*

*(P12)*

*The fever and the pain medication did not help, I still had fever and headache, so they explained me how to take them and I was relieved.*

*(P1)*

Some received advice about how to minimise the risk of contagion:

*I received instructions on how to clean the house, how to disinfect so as not to spread the infection, etc. But honestly I didn’t listen to them, nor was I interested. You hear what you want to hear, and not what the other person says.*

*(P7)*

Some of the patients learned strategies to reduce stress:

*I have a tendency to have a bit of nerves, even a little anxiety. I remember one of the nurses telling me to try mindfulness.*

*(P1)*

Others applied cognitive restructuring to obtain better emotional well-being:

*To think positively, that the anger that I was focusing on specific people who hadn’t done things right... And they told me that what I had to do was take care of myself and set aside the rest. That I shouldn’t think negatively.*

*(P2)*

Most participants reported finding a space of mental space of calm and confidence that enabled them to move forward and take care of themselves.

*A nurse told me that in terms of morale, I had to be strong, or that I wouldn’t get over it. That I should force myself to eat. I hardly ate anything... That I should eat meals, even if it was just a little.*

*(P7)*

In this sense, the participants established routines that contributed to their well-being:

*The issue of sports I took seriously. I continued to live a normal life to the extent that my body allowed it.*

*(P5)*

Most participants reported that the nursing telecare helped them to deal with the situation.

*They ask you how you are and, depending on what you say, they tell you, “Do this, do that. Write down such-and-such a symptom so you can tell me tomorrow”. And in the end, you see that it helps you.*

*(P8)*

The information received in the nursing phone calls allowed participants to take care of their own basic physical and psychological needs. 

#### 4.3.2. Emotional Support

The data show that the nursing intervention went beyond a simple phone call to monitor physical symptoms. It was also a means for the person to feel heard and to receive encouragement.

*The symptom checklist can be done by a computer, but emotional support is what people can provide. It’s this more empathetic part, more about putting yourself in the other person’s shoes. Not everyone can do that, and I think it’s very good.*

*(P7)*

Others said reassuring messages had been important for them:

*It especially helped me a lot psychologically when they reassured me and when they said “Don’t worry. You’ve have been without symptoms for a lot of days. Don’t stop doing this [aspect of the treatment], but you’re almost there”.*

*(P3)*

Other participants explained that they felt reassured by knowing that they could call the nurses if their condition worsened.

*The role of support and knowing that if I really had a problem, there would be someone there. If today I know that I don’t have a problem, I know that I have a phone in case I’m not okay tomorrow. Or that if tomorrow when they call me, I can tell them, “Look I’m worse, I can’t breathe”. This is a relief. Because you know there’s someone, at time of such solitude, when you’re locked up at home.*

*(P13)*

In addition, some participants mentioned the importance of feeling that they had been treated as individuals.

*The first few days you’re scared. I was scared, causing myself a kind of anxiety, thinking that I wouldn’t be able to breathe. You start imagining things. In this sense it gives you confidence that they [the nurses] reassure, advise you.*

*(P14)*

Feeling supported by their nurses was one of the main experiences reported by participants.

*To change your mindset, to help me get out of my self-destructive spiral, so to speak. What I remember most isn’t so much what they [the nurses] asked me about symptoms, but the small conversations about everyday life. You’re a person.... and that made me see that I was a person first and a patient second.*

*(P7)*

However, some participants felt that they had not received enough support for their emotional needs from nurses.

*What would have been necessary was a call from time to time to see how I was. Because I wouldn’t have been so stressed out at least in that sense because you feel supported and the professional is concerned that you don’t get worse.*

*(P3)*

The majority of participants felt emotionally supported, even though the intervention was not carried out face-to-face.

#### 4.3.3. Personal Commitment

Participants also described that nurses helped them commit to feeling better.

*I did what the nurses were telling me, to think positively. I got involved. Something clicked. I don’t know who called me, but something clicked, “I have to take care of myself”. And from that day, I started to feel better.*

*(P2)*

It also helped them to identify the causes of stress.

*I was able to identify my stress and set it aside. Not struggle against it. Now I’m not afraid.*

*(P11)*

Some participants reported the importance of feeling active in social life and work, despite the situation:

*Since I am self-employed, I kept working on all the projects I had. And I kept working as many hours as I could when I was mentally better, and they [the nurses] supported me in that.*

*(P11)*

Finally, some reported a personal commitment toward self-care.

*I’ve learned a lot about myself and how to take care of myself. I hope to continue like this because now I feel able to face the day-to-day with a better attitude.*

*(P2)*

## 5. Discussion

The present study explored the experiences of COVID-19 patients receiving telenursing from primary care nurses during the first wave of the pandemic. The participants reported their physical symptoms, their emotional and social difficulties, and their difficulties in receiving accurate information about COVID-19. Also, the participants described strategies for overcoming challenges and the role of nurses in terms of self-care, feeling emotionally supported and making a personal commitment. 

At a physical level, most of our participants had mild or moderate symptoms, which is unsurprising since we excluded patients who had to be admitted to a hospital. Participants were able to describe their symptoms to their nurses and receive reassurance, an important finding given that another study reports a lack of understanding of symptoms as a major stressor for patients in isolation for COVID-19 [32]. A disadvantage of virtual care is the lack of direct physical contact, which may limit the nurses’ ability to perform certain clinical assessments, such as auscultations or detailed physical examinations. Coordination with the physician was essential to provide face-to-face care to patients requiring a physical examination [33] (telenursing COVID-19 iherph). Participants reported medication explanations received but did not include self-medication as self-care, possibly symptom management and concerns were addressed with adequate care and support Kord [9]

Most participants reported feeling vulnerable and demoralised or being afraid of infecting family members [13]. They also felt rejected, stigmatised for possibly having infected others and guilty [18]. The participants reported that talking with their nurses helped them manage these feelings. These results suggest that telenursing can be used to help patients handle troubling social situations in addition to physical symptoms [34] 

We did not ask participants about the information that they received about COVID-19, but many of them complained that it was inadequate. Unlike the other challenges, participants did not mention the ways in which they overcame this problem, suggesting perhaps that this problem was not sufficiently addressed in nursing telecare. Active listening and effective questioning techniques are key enablers in virtual care. By attentively listening to patients and asking the right questions, healthcare providers can gather essential information, understand patients’ concerns, and provide appropriate support and guidance. These skills help establish a strong therapeutic relationship even in a virtual setting, enhancing the quality of care delivered remotely [35]. 

This is an interesting area for future research, given that early studies showed that the general population’s level of knowledge about COVID-19 in different places around the world was only moderate. Another finding of the study was the lack of information caused mistrust, anger and increased psychological distress in our participants, as with other studies [9,18]. Importantly, this lack of information could be due to the high frequency of changes in protocols for treating COVID-19, as we have shown [15]. Severe conditions caused by COVID-19 have caused a complex application of remote care [36]. 

The strategies participants used to overcome COVID-19 in home isolation were learning self-care, accepting emotional support from their nurses and making a personal commitment to improve. 

We have shown that telenursing supported participants’ ability to care for themselves and to deal with the stress caused by isolation and the uncertainty of their clinical course. Nurses gave guidelines for understanding symptoms and providing self-care. Through self-care, the participants were able to meet their basic needs of eating, sleeping, rest and physical activity, as seen in other studies [37]. We thus suggest that through telenursing, nurses can provide information about self-care, successfully answer questions and motivate people to change their behaviour. Prior research has already shown that nurses provide this kind of support through in-person care; our study suggests that these activities also take place in telenursing, extending findings about telecare in work such as that by Hincapié et al. [38].

Our study also shows the importance of interactions between patients and professionals in virtual environments. Receiving psychological support from nurses is shown to be very effective in studies such as that by Rajkumar [13]. This support is achieved with empathy and humanisation in care [8]. In particular, giving guidelines to reduce stress, promote a positive mental attitude and encourage cognitive restructuring for behavioural modification can reduce the feeling of solitude caused by medical isolation [39]. In this sense, our findings support the claim that patient-centred care can be provided through telenursing [40] and confirm that telephone monitoring is reliable for mild COVID patients. 

Although the initial telenursing intervention may have been driven by urgency, it is valuable to consider the existing literature and explore the application of more robust theories and techniques in future telenursing interventions. In this sense, a specific theoretical model of self-care and well-being was not used as the basis for the intervention [5]. However, it is important to note that there is literature supporting the use of cognitive-behavioral and motivational theories and techniques in the assessment of patients’ perceptions of their health status. These theories and techniques can provide a solid framework for understanding and addressing the psychological and emotional aspects related to health care, including in a telenursing context, and thus achieve the objectives proposed by the professionals.

### Limitations

This study has some limitations. One is that our results can only be transferred to similar clinical contexts. The sample is small and therefore is not representative of all COVID patients with a similar profile. This study can be a launch point, useful for comparison with larger studies in other contexts, to identify best practices in telenursing intervention and could reinforce the consistency of the present study. It is also important to note that, as a qualitative study, it was not possible to quantify the effect of experiences on the COVID patients in home isolation since the research only allowed for the description of experiences of care and participation in the telenursing intervention. Finally, this study includes only the perspective of patients. Future research in this area should be continued on a larger group of patients, and especially the perspective of nurses providing telecare should be known so that they can be compared and contrasted with the perspective of patients.

## 6. Conclusions

This research provided insight into the experiences of COVID-19 patients who received telenursing while they were in home isolation in Catalonia (Spain) and the importance of a telenurse in providing individualised care. The patients reported having achieved strategies to improve their physical, psychological and situational well-being despite the unprecedented situation. We showed that telenursing is a useful resource in caring for COVID-19 patients at home because it allows a patient-centred approach despite the fact that patients and professionals do not meet face-to-face. The Catalan health system and others could consider the use of videoconferencing to reinforce the model of person-centred care in telenursing. The advantages and drawbacks of telenursing should be kept in mind for future health crises, especially those involving infectious diseases. 

### Implications for Nursing Policy

The COVID-19 crisis has placed unprecedented demands on health and social care services worldwide. Because of this, public emergency primary care nurses have played a central role in the management of mild to moderate COVID patients. The pandemic has forced healthcare professionals to make progress in the digitalisation of healthcare. Notably, the telenursing intervention provided two benefits. First, despite being designed quickly, it allowed the management of symptoms and self-care, confirming that telephone monitoring is reliable for mild COVID patients. Second, digital solutions are central to a patient-centred care model, and our results make it possible to reflect on the factors that encourage personal commitment in patients. Hence, the study provides results that can improve clinical decision-making and expand telenursing as a dynamic and optimal resource for patient care. This knowledge can help nurses, other health professionals and policymakers to launch and lead e-health projects and explore new forms of remote care, which could be employed for treating other infectious diseases and for treating patients with limited mobility. 

## Figures and Tables

**Table 1:** Interview guide.

I would like you to think for a bit about how things were for you when you were in home isolation because of COVID. - What was it like when you found out about the diagnosis? - How did you feel physically? What did you do to improve your physical well-being? - Could you tell me something about your emotional well-being during the period that you were in isolation because of COVID? How did you deal with your emotional state? - Could you tell me something about your social life while you were in isolation because of COVID: work, friends, relationships with others, personal activities and family roles? How did you handle this situation? I would also like to talk about your experience with the telenursing follow-up that you received. - Did they give you information about self-caring? - How did the follow-up influence how you managed your emotions? - How would you evaluate self-care activities in general?

**Table 2:** Participants’ sociodemographic characteristics.

Variables	*n*	%
**Distribution by gender**		
male	5	36%
female	9	64%
total	14	100%
**Distribution by age**		
20–29	2	14%
30–39	5	36%
40–49	0	0%
50–59	7	50%
total	14	100%
**Marital status**		
single	6	43%
married	5	36%
divorced	3	21%
other	0	

## Data Availability

Not applicable.

## References

[1] Agencia Salut Publica de Catalunya (2020). NFORME-TECNIC-1-COVID-19-19032020.pdf. https://coronavirus.coib.cat/wp-content/uploads/2020/05/INFORME-TECNIC-1-COVID-19-19032020.pdf.

[2] Bokolo A.J. (2021). Application of telemedicine and eHealth technology for clinical services in response to COVID-19 pandemic. Health Technol..

[3] Kapoor A., Guha S., Kanti Das M., Goswami K.C., Yadav R. (2020). Digital healthcare: The only solution for better healthcare during COVID-19 pandemic?. Indian Heart J..

[4] Zheng S., Yang L., Zhou P., Li H., Liu F., Zhao R. (2021). Recommendations and guidance for providing pharmaceutical care services during COVID-19 pandemic: A China perspective. Res. Soc. Adm. Pharm..

[5] Souza-Junior V.D., Mendes I.A.C., Mazzo A., Godoy S. (2016). Application of telenursing in nursing practice: An integrative literature review. Appl. Nurs. Res..

[6] Schlachta-Fairchild L., Varghese S.B., Deickman A., Castelli D. (2010). Telehealth and Telenursing Are Live: APN Policy and Practice Implications. J. Nurse Pract..

[7] Imlach F., McKinlay E., Middleton L., Kennedy J., Pledger M., Russell L., Churchward M., Cumming J., McBride-Henry K. (2020). Telehealth consultations in general practice during a pandemic lockdown: Survey and interviews on patient experiences and preferences. BMC Fam. Pract..

[8] Halcomb E., Williams A., Ashley C., McInnes S., Stephen C., Calma K., James S. (2020). The support needs of Australian primary health care nurses during the COVID-19 pandemic. J. Nurs. Manag..

[9] Kord Z., Fereidouni Z., Mirzaee M.S., Alizadeh Z., Behnammoghadam M., Rezaei M., Abdi N., Delfani F., Zaj P. (2021). Telenursing home care and COVID-19: A qualitative study. BMJ Support. Palliat. Care.

[10] American Academy of Ambulatory Care Nursing (AAACN) (2018). Scope and Standards of Practice for Professional Telehealth Nursing.

[11] Arnaert A., Girard A., Craciunas S., Shang Z., Ahmad H., Debe Z., Demyttenaere S. (2022). Patients’ experiences of telenursing follow-up care after bariatric surgery. J. Clin. Nurs..

[12] Yliluoma P., Palonen M. (2020). Telenurses’ experiences of interaction with patients and family members: Nurse–caller interaction via telephone. Scand. J. Caring Sci..

[13] Rajkumar R.P. (2020). COVID-19 and mental health: A review of the existing literature. Asian J. Psychiatr..

[14] Romeu-Labayen M., Tort-Nasarre G., Alvarez B., Subias-Miquel M., Vázquez-Segura E., Marre D., Galbany-Estragués P. (2021). Spanish nurses’ experiences with personal protective equipment and perceptions of risk of contagion from COVID-19: A qualitative rapid appraisal. J. Clin. Nurs..

[15] Tort-Nasarre G., Alvarez B., Galbany-Estragués P., Subías-Miquel M., Vázquez-Segura E., Marre D., Romeu-Labayen M. (2021). Front-line nurses’ responses to organizational changes during the COVID-19 in Spain. A qualitative rapid appraisal. J. Nurs. Manag..

[16] Arora T., Grey O., Östlundh L., Lam K., Omar O., Arnone D. (2022). The prevalence of psychological consequences of COVID-19: A systematic review and meta-analysis of observational studies. J. Health Psychol..

[17] Akbarbegloo M., Sanaeefar M., Majid P., Mohammadzadeh M. (2021). Psychosocial care experiences of patients with COVID-19 at home in Iran: A qualitative study. Health Soc. Care Community.

[18] Brooks S.K., Webster R.K., Smith L.E., Woodland L., Wessely S., Greenberg N., Rubin G.J. (2020). The psychological impact of quarantine and how to reduce it: Rapid review of the evidence. Lancet.

[19] James S., Ashley C., Williams A., Desborough J., McInnes S., Calma K., Mursa R., Stephen C., Halcomb E.J. (2021). Experiences of Australian primary healthcare nurses in using telehealth during COVID-19: A qualitative study. BMJ Open.

[20] Moltó-Puigmartí C., Segur-Ferrer J., Berdún Peñato J., Piera Jiménez J., Estrada-Sabadell M.D., Vivanco-Hidalgo R. (2022). Evaluación de la Teleconsulta en Atención Primaria.

[21] Turale S. (2020). A brief introduction to qualitative description: A research design worth using. Pacific Rim Int. J. Nurs. Res..

[22] Colorafi K.J., Evans B. (2016). Qualitative Descriptive Methods in Health Science Research. Health Environ. Res. Des. J..

[23] Latorre A., Del Rincón D., Arnal J. (1996). Bases Metodológicas de la Investigación Educativa.

[24] Maykut P., Morehouse R. (1999). Investigación Cualitativa: Una Guia Práctica y Filosófica.

[25] Morse J., Field P. (1995). Qualitative Research Methods for Health Professionals.

[26] O’Reilly M., Parker N. (2013). ‘Unsatisfactory Saturation’: A critical exploration of the notion of saturated sample sizes in qualitative research. Qual. Res..

[27] Johnson J.L., Adkins D., Chauvin S. (2020). A review of the quality indicators of rigor in qualitative research. Am. J. Pharm. Educ..

[28] Braun V., Clarke V. (2014). What can “thematic analysis” offer health and wellbeing researchers?. Int. J. Qual. Stud. Health Well-Being.

[29] Guba E., Lincoln Y. (1994). Handbook of Qualitative Research.

[30] Green J., Thorogood N. (2013). Qualitative Methods for Health Research.

[31] Tong A., Sainsbury P., Craig J. (2007). Consolidated criteria for reporting qualitative research (COREQ): A 32-item checklist for interviews and focus groups. Int. J. Qual. Health Care.

[32] Mucci F., Mucci N., Diolaiuti F. (2020). Lockdown and Isolation: Psychological Aspects of Covid-19 Pandemic in the General Population. Clin. Neuropsychiatry.

[33] Heo H., Lee K., Jung E., Lee H. (2021). Developing the first telenursing service for COVID-19 patients: The experience of South Korea. Int. J. Environ. Res. Public Health.

[34] Mostafaei A., Sadeghi-Ghyassi F., Kabiri N., Hajebrahimi S. (2022). Experiences of patients and providers while using telemedicine in cancer care during COVID-19 pandemic: A systematic review and meta-synthesis of qualitative literature. Support. Care Cancer.

[35] Russell A., De Wildt G., Grut M., Greenfield S., Clarke J. (2022). What can general practice learn from primary care nurses’ and healthcare assistants’ experiences of the COVID-19 pandemic? A qualitative study. BMJ Open.

[36] Moore M.A., Munroe D.D. (2021). COVID-19 Brings about Rapid Changes in the Telehealth Landscape. Telemed. e-Health.

[37] Cole-King A., Dykes L. (2020). Wellbeing for HCWs during COVID19. https://www.lindadykes.org/covid19.

[38] Hincapié M.A., Gallego J.C., Gempeler A., Piñeros J.A., Nasner D., Escobar M.F. (2020). Implementation and Usefulness of Telemedicine During the COVID-19 Pandemic: A Scoping Review. J. Prim. Care Community Health.

[39] Pedrosa A.L., Bitencourt L., Fróes A.C.F., Cazumbá M.L.B., Campos R.G.B., de Brito S.B., Simões e Silva A.C. (2020). Emotional, Behavioral, and Psychological Impact of the COVID-19 Pandemic. Front. Psychol..

[40] Tebeje T.H., Klein J. (2021). Applications of e-Health to Support Person-Centered Health Care at the Time of COVID-19 Pandemic. Telemed. e-Health.

